# The Lake Wobegon Effect—Where Every Medicare Advantage Plan Is “Above Average”

**DOI:** 10.1001/jamahealthforum.2022.4320

**Published:** 2022-10-07

**Authors:** Joan M. Teno, Claire Ankuda

**Affiliations:** Department of Health Services, Policy, and Practice, School of Public Health, Brown University, Providence, Rhode Island; Behavioral and Policy Sciences Department, RAND Corporation, Arlington, Virginia; Brookdale Department of Geriatrics and Palliative Medicine, Icahn School of Medicine at Mount Sinai, New York, New York

That’s the news from Lake Wobegon, where all the women are strong, all the men are good looking, and all the children are above average.

Garrison Keillor

A recurring segment on the Prairie Home Companion, a long-running weekly public radio variety show created and hosted by Garrison Keillor, characterized all the children of the fictional town of Lake Wobegon as “above average.” Although this statistically impossible scenario may be unremarkable in a whimsical radio program, it would seem surprising in ratings for insurance plans. However, for Medicare Advantage (MA) plans in the MA Quality Bonus Program (QBP)—a program that results in higher payments for “above average” plans with a 4- or 5-star rating—as the QBP has evolved over time, nearly all Medicare beneficiaries are now enrolled in MA plans that are rated “above average.”

Is it true that so many MA plan enrollees are now part of plans deemed “above average” when in 2012 only 28% were? How did this happen?

The Affordable Care Act in 2010 mandated the creation of the 5-star rating system for MA plans. Under this system, plans receiving a 4- or 5-star rating would be given a 5% or 10% increase in their benchmark, allowing such plans to increase their contract bid and resulting in increased payments. As of February 2020, among MA contracts with star ratings, 83% of MA beneficiaries were enrolled in plans that received a bonus, resulting in $6 billion per year in QBP costs.^1^ By 2022, these costs increased to $10 billion. In part, this striking change in the share of recipients in plans receiving a bonus was not from improvements in the quality of care, but from policies that allow MA plans to consolidate contracts and merge lower-rated MA plans with contracts of plans that have higher ratings. The Medicare Payment Advisory Committee (MedPAC) estimates that as a result of contract consolidation, 37% of Medicare beneficiaries were “upcoded” from contracts with plans that had a rating lower than 4 stars to having a plan with a rating of 4 or 5 stars.

By 2030, MA is on track to care for 69% of Medicare beneficiaries.^2^ Because MA beneficiaries are older adults, the stakes are high in getting quality measurement right. In theory, the value-based financing of MA reverses the incentives from doing more to providing cost-effective, high-quality, and equitable care. These same incentives could also motivate plans to achieve cost savings by just doing less: by denying beneficial treatments, requiring high co-payments for more costly services, or offering inadequate networks of clinicians and health care facilities. To date, the black box of MA quality has not received appropriate due diligence, especially for frail, older people and those with serious illness who have the greatest health care needs.

The one resource that consumers have to help them choose MA plans is the Medicare Plan Finder website from the US Centers for Medicare & Medicaid Services (CMS). However, the information reported on this website is based not only on the local MA plan that the consumer is choosing but rather on the contract number with which the local MA plan is associated. A handful of parent companies, including Humana and UnitedHealthcare, run the vast majority of MA plans. These parent companies negotiate contracts, and each contract involves local plans offered to consumers. The local plans included in the contract can vary with respect to networks of clinicians and health facilities, as well as benefit structures, such as yearly deductibles. Moreover, the CMS allows parent companies to consolidate plans into contracts as they wish, regardless of geography. For example, a UnitedHealthcare plan in Rhode Island with contract #H1944 is ranked based on data from 17 different plans across Massachusetts, Rhode Island, Vermont, Pennsylvania, and New Hampshire.

The current system of rating MA plans does not allow for meaningful comparisons. Although the Federal Rule for MA published in 2018 discussed options, it is time to reconsider the options especially in the case when the contract covers noncontiguous states. Reporting at the level of individual plans may not be possible for all quality measures, given that the small size of some plans would result in measurement error. However, at the very minimum, information on the Medicare Plan Finder website could make it clear to consumers that information on quality may not adequately inform their decision regarding care in a specific local plan.

Wennberg and colleagues have documented the geographic variation in the health care that US patients receive (such as in rates of hospital use and mortality in 2 different cities in New England), outlining the variation in utilization and quality of care that is local. The genesis of this research was based on data that tonsillectomy rates in Waterbury Center, Vermont, were 20% compared with a rate of 60% in the adjacent town of Stowe. Utilization and quality of care are local—yet the CMS Medicare Plan Finder website provides information about MA plans at the contract level that may cover the quality of care based on noncontiguous states and diverse plans. Based on a robust body of research showing geographic variation in health care, the quality of care in Pennsylvania is unlikely to reflect the quality of care in Rhode Island.

Concerns of the CMS about measurement error with small plans are important, but it is time to consider solutions that balance the need to minimize this error with the need to provide meaningful information to consumers. The agency could classify which measures can be reported at the contract level vs those that need to apply to a smaller geographic region to provide accurate information for consumers. Although some aspects of quality measures are process measures about the oversight of centralized functions (such as timeliness of appeals) that are likely consistent across contracts, for other measures, as Wennberg and colleagues have demonstrated, quality of care is often based on local networks of clinicians and hospitals and other health care facilities. One possible approach to reporting of locally sensitive measures could involve collecting 8 quarters of data to achieve sufficient reliability to allow comparisons at the plan level or simply informing consumers that quality measures at the plan level are not available.

Given the growth of MA, the need for Congress to create a process to review and update the QBP program for MA plans is urgent. Congress already mandated MedPAC to report on the quality of dual-eligible special needs plans, which are specialized MA plans that are designed to meet the specific needs of beneficiaries dually eligible for Medicare and Medicaid. An increasing number of frail and older individuals are receiving care as part of dual-eligible special needs plans and other special needs plans. And yet, as MedPAC reported in their March 2022 report,^3^ the performance data that MA plans report provide “limited insight” on how well dual-eligible special needs plans perform compared with other plans that serve dual-eligible beneficiaries.

The current system for rating the quality of MA plans does not allow consumers to make meaningful comparisons. The millions of US seniors faced with choosing an MA plan deserve to know if a given plan is truly above average—or if a favorable rating might be a fictional entity, not unlike Lake Wobegon’s ubiquitously above-average children.
